# Dynamic Stiffness of Bituminous Mixtures for the Wearing Course of the Road Pavement—A Proposed Method of Measurement

**DOI:** 10.3390/ma13081973

**Published:** 2020-04-23

**Authors:** Krzysztof Robert Czech, Wladyslaw Gardziejczyk

**Affiliations:** 1 Department of Geotechnics and Structural Mechanics, Bialystok University of Technology, 15-351 Bialystok, Poland; 2 Department of Construction and Road Engineering, Bialystok University of Technology, 15-351 Bialystok, Poland; w.gardziejczyk@pb.edu.pl

**Keywords:** dynamic stiffness, wearing course, bituminous mixtures, test stand

## Abstract

Stiffness is an important mechanical characteristic of asphalt mixtures used in the wearing course. It is one of the determining factors in the generation of tyre/road noise. The dynamic stiffness of the upper layer of the road surface depends on the physical and mechanical properties of the materials it is composed of, and traffic load. Determination of dynamic stiffness, both in laboratory conditions and in situ, requires consideration of many other factors. Tests of dynamic properties of road surfaces in field conditions are most often conducted with the help of modal hammers. Impulse excitation results are usually less accurate than those in the application of modal exciters. The test stand was constructed, comprising a tripod, 32-channel and 24-bit data acquisition system, exciter, signal amplifier, impedance head, single-axis piezoelectric accelerometers and a stinger. The test stand and the proposed method of measuring dynamic stiffness do not require the determination of the resonance frequency of the tested specimen and can be used both on various types of bituminous mixtures of varying shape and dimensions, as well as directly on the upper surface of the wearing course of bituminous pavements. The test results showed that the type of bituminous mixture used in the wearing course significantly affects its dynamic stiffness. The dynamic stiffness level of asphalt concrete, stone mastic asphalt and porous asphalt layers was determined to be similar. The addition of rubber granulates significantly reduced its rigidity, which is very beneficial from the point of view of reducing the tyre/road noise.

## 1. Introduction

The study of the relationship between a given force in dynamic terms and the material’s response, expressed as displacement, velocity or acceleration, is a complex issue. This is a particularly difficult problem for bituminous composites used in the wearing course of road pavement. The stiffness of the upper layer of the road surface depends on the physical and mechanical properties of the materials it is composed of, and the operating conditions and traffic load. The determination of dynamic stiffness, both in laboratory conditions and on real surfaces, requires consideration of many other factors. In the case of laboratory tests, it is necessary to pay attention, inter alia, to the dimensions of the samples tested and the temperature. In tests on real road surfaces, the thickness of the wearing course, the characteristics of the materials and the thickness of the binder layer and base course are of great importance. In both cases, the method of dynamic load implementation has a significant impact.

In the case of traffic-induced vibrations, frequencies at 1 Hz are generated as a result of the change of surface deflection due to the weight of vehicles’ non-oscillating movements. Slightly higher vibration frequencies, of the order of 3–4 Hz, are generated from passing unloaded vehicles as a result of vehicles’ body fluctuations on their suspension and passing tractors. According to Hao [1] vibration frequencies at about 0.6–0.7 Hz correspond to the vehicle body-bounce. Higher frequencies of 10–20 Hz are generated during vehicle wheel-hopping. The most intense vibrations in bituminous surfaces are usually generated during the passage of multi-axle heavy vehicles. For example, according to the results of the tests presented in [2], in the case of a two-axle lorries travelling on a bituminous surface with different construction at a speed of 48 km/h, the dominant vibration frequencies range from 22.2 Hz to 30.4 Hz. With the increase in vehicle travel speed to the speed in force on city ring roads, expressways and motorways, the dominant vibration frequencies will certainly increase. However, there is no conclusive data on this subject in literature. According to DIN 4150-3 [3], from the point of view of the possible impact of traffic-induced vibrations propagated in the ground and transmitted on buildings, vibrations with frequencies from 1 to about 100 Hz are of significant importance. In general, in dynamic diagnostics of buildings, we usually encounter vibrations in the frequency band from 4 Hz to 250 Hz (according to BS 7385-2 [4] and BS 5228-2:2009 [5]). Only in exceptional cases, such as vibrations originating from man-made sources propagated in hard ground, will frequencies up to 1000 Hz appear [4].

The most commonly used parameter that characterizes the stiffness of bituminous mixtures is the frequency–response function, defined as a dynamic stiffness or a complex modulus. The dynamic stiffness is determined in the laboratory using inter alia four-point bending test and an indirect tensile test, according to the EN 12697-26 standard [6]. The four-point bending test is performed at a temperature of 20 °C and for the frequencies of 0.1, 0.2, 0.5, 1, 2, 5, 8 and 10 Hz. The indirect tensile test is applicable to cylindrical specimens of different diameters and thickness. After conditioning the specimen, five pulse loads are applied on the specimen, recording the variation of the applied load and the horizontal diametral deformation. Relevant literature presents, in detail, the results of tests and analyses in relation to dynamic stiffness, expressed as a mechanical impedance, an energy dissipation or a complex modulus (E*), depending on the type of mix, frequency, load and temperature [7,8,9,10,11,12].

The tyre/road noise depends on type and speed of vehicles and on the road pavement characteristics. Porous asphalt (PA) and thin asphalt layers (e.g., BBTM—thin asphalt concrete, SMA LA—low-noise stone mastic asphalt) are defined as the low noise pavements. In the case of these surfaces, their noisiness is determined by the air void content, maximum aggregate size, thickness of layers and their exploitation lifetime. The third generation of low noise pavements are poroelastic road surfaces (PERS). They are characterized by an air void content from 20% to 40%, at least a 20% proportion of crumb rubber by weight of mixes and higher flexibility than asphalt concrete (AC) and SMA mixes. Crumb rubber could be incorporated into the mixtures by dry and wet processes. In a dry process, crumb rubber replaces some of the fraction aggregates in the mixture. In the wet process, it is added to bitumen before mixing with the aggregates.

However, testing rigidity of poroelastic mixtures based on standard laboratory method poses a problem. In the PERSUADE project [13], the results of the examination of the complex modulus E* were presented. It was found that the typical asphalt concrete mix had about 150–1500 times higher dynamic moduli values than the poroelastic mixtures with no hard aggregates and about 20–200 times higher than poroelastic mixtures with 82% of hard aggregates. In indirect tensile tests, the poroelastic samples, softer than asphalt concrete samples, did not completely deform and break after loading, but had dents at both the top and bottom loading platens.

A device dedicated to the research of dynamic stiffness, the Dynamic Shear Rheometer (DSR), helped the authors of [14] to examine bituminous samples in the frequency range from 0.01 Hz to 25 Hz, at temperatures: +4 °C, +20 °C, +40 °C.

Much more opportunities in regard to applied loads are provided by a servo-hydraulic fatigue testing machines (for example, INSTRON, MTS, ZWICK). In their case, it is possible to apply the loading of the samples with a force of 100 kN or higher at frequencies reaching 10 Hz, in some testing devices up to 40 Hz, and very rarely up to 100 Hz. They can also be equipped with thermal chambers.

Tests for dynamic properties of specimens using this type of fatigue machine were carried out, among others, in work [15], in which the effect of moisture conditioning on dynamic modulus of porous asphalt was studied. MTS Landmark Servohydraulic Test Systems type 370.10 equipped with a temperature chamber was used for applying loads to samples. The tests were carried out at six frequency levels (0.1 Hz, 0.5 Hz, 1.0 Hz, 5 Hz, 10 Hz and 25 Hz) and for five different temperatures (−10 °C, 4 °C, 21 °C, 37 °C and 54 °C).

The presented data show that, in laboratory tests of asphalt mixtures, the dynamic parameters of samples are not generally determined for frequencies exceeding 25 Hz, despite the fact that DIN EN 12697–26 [6] recommends determining the dynamic stiffness in the frequency range as wide as possible. In contrast, the frequency range of sounds audible to humans covers the range from about 16 Hz up to about 20 kHz [16].

In situ studies of dynamic properties of road pavements conducted so far are most often based on impulse excitation applied by means of various types of modal impact hammers and registration of motion response using one or several transducers located on the surface of the wearing course.

In this type of research, depending on adopted boundary conditions and whether motion is expressed as displacement, velocity or acceleration, the so-called “structural response ratios” are determined (for example: dynamic stiffness, dynamic compliance, mobility, mechanical impedance, accelerance, effective mass) [17].

An example of this type of research is described, among others, in [18]. To measure mechanical impedance [Ns/m], a modal impact hammer was used to hit the impedance head and an accelerometer glued to the pavement at a distance of 10 cm from the impedance head. Six attempts were made in each case. The measurements were carried out both on the Poro-Elastic Road Surface (PERS) (in 5 different locations) and in laboratory conditions on PERS boards placed on a concrete slab (1 m × 1 m in a horizontal projection). However, the work in [18] does not provide more detailed information regarding data acquisition parameters and research equipment used, which makes it difficult to assess the reliability of the presented results.

The authors of [19] indicate the possibility of using the “The PiScan Probe” device to measure dynamic properties of bituminous mixtures. It is composed of a modal impact hammer and two accelerometers with weights ensuring adequate pressure of the accelerometers to the wearing course, which may be at a distance of 15 cm or 30 cm during the tests. The accelerometers are connected to each other by a special frame. During the tests, the distance between the point of impact with the modal hammer and the first and second accelerometer was 30 cm each (SAWS test). In the case of resonance tests, modal impact hammer strikes were carried out at a distance of 7.5 cm from the accelerometer mounting point (MP1). Similar to the work in [18], the parameters of the modal hammer, accelerometers and data acquisition parameters used in the study are not given.

The dynamic properties of the material are much easier to examine in field conditions using modal impact hammers than with the use of vibration exciters. Unfortunately, as in the case of experimental modal analyses of building structures, machines and vehicles, the results obtained with impulse excitation are usually not as accurate (reliable) as in the case of using modal exciters [20,21].

The purpose of the research is to develop an experimental test stand that will allow measurement of the dynamic stiffness of bituminous mixtures in laboratory conditions and dynamic stiffness of bituminous road pavements in field conditions. The article presents a test stand and the preliminary results of laboratory tests.

## 2. Methods and Test Stand

The development of the concept of the test stand for use in testing the dynamic stiffness of mixtures used in road construction with different characteristics (from standard asphalt, through layers of porous asphalt, to poroelastic pavements), based on vibration exciter, was preceded by the analysis of work carried out by other researchers. Some of them are mainly based on the provisions of the EN 29052-1 standard [22] and concerned solutions used in floating floors.

According to EN 29052-1 [22], apparent dynamic stiffness per unit area of test specimen *s_t_′* (1) or dynamic stiffness per unit area of resilient material *s′* is determined based on the total mass per unit area *m_t_′* [kg/m^2^] and the extrapolated resonance frequency *f_t_* [Hz] determined from the recorded frequency spectra (frequencies corresponding to peak values in the graph or phase shift between excitation and vibration signal).

*s_t_*′ = 4 π^2^*m_t_*′ *f_t_*^2^ (1)

During the test, both the load plate and the massive base plate may be subject to vibration. The excitation can take the form of sinusoidal, white noise or pulse signals. In the case of excitation with a sinusoidal signal, the resonance frequency is determined by varying the frequency of excitation, while keeping the excitation force constant (so-called swept sinusoidal excitation). In the case of excitation with white noise or pulse signals in EN 29052-1 [22], it is advised to apply the provisions of ISO 7626-2 [23].

However, the standard [22] does not specify how the excitation of vibrations, the connection of the source of vibrations with the tested sample or the base plate should be carried out. It is also pointed out that the impact of the total load on the test specimen to the level of determined dynamic stiffness can be from 10% to 20% (the difference in the case of static load at 2 kPa and in the case of factual absence of static load on the specimen). Moreover, according to this standard, the determination of dynamic stiffness per unit area of resilient materials can only be carried out for materials working under load in the range from 0.4 kPa to 4.0 kPa.

The assumptions of the standard EN 29052-1 [22] were used by the authors of the works [24,25] in determining the dynamic stiffness of bituminous materials, by the so-called “resonance method”. The only difference was that the samples tested and the steel load plate had the shape of a cylinder, and not a prism. The driving force was applied as swept sinusoidal excitation. To generate vibrations, an exciter attached to the tripod by using four springs was applied. To record the exciting force and resulting acceleration response of the specimen submitted in the test to driving force a vibration exciter, signal amplifier, impedance head and vibration analysers were used.

Similarly, by using an exciter, a stinger of high longitudinal stiffness and sufficient flexibility in other directions, and an impedance head mounted to the tested sample, it is possible to determine its dynamic stiffness. This approach was also applied in [24]. In contrast to the resonance method, a 14-mm diameter circular plate was placed between the impedance head and tested sample instead of the steel load plate transmitting the load to the entire upper surface of the sample. A frequency random excitation of vibrations was used in the test in place of sinusoidal excitation. The measurement carried out in this way was called the “non-resonant method”. Given the maximum aggregate grain size in asphalt mixes (up to 11–14 mm), the assumed loading diameter seems to be insufficient. Also, the work in [24] does not specify what kind of random excitation was generated (white noise/pink noise), and what was the level of vibration amplitude, or the level of amplification of the generated signal expressed in volts (V). The authors of [24] rightly noted that, unlike the resonance method, the above method of testing dynamic stiffness is more appropriate in testing real road surfaces.

Bearing in mind the above analysis, it was found that the method of dynamic load implementation needs to be clarified, in particular: the method of load transfer from the exciter via the so-called stinger on the tested material, surface area of the load transferring element from the exciter to the tested sample or wearing course of the pavement, static and dynamic stress in the test specimen or pavement directly under the contact element.

When creating the test stand of Bialystok University of Technology, the assumption was made that it should provide the possibility of performing the dynamic stiffness measurements of bituminous samples with very different characteristics under laboratory conditions, as well as on real road surfaces. It was also assumed that dynamic stiffness measurement based on broadband excitation will be carried out using the resonance and/or non-resonant method. The following equipment was selected for the development of the stand, listed in the technical inventory of the Department of Geotechnics and Structural Mechanics at the Bialystok University of Technology (BUT):

- 32-channel and 24-bit data acquisition hardware type SCADAS Recorder from SIEMENS with 130 dB dynamic range and signal to noise ratio—minimum 106 dB;
- induction exciter TIRA TV 51144IN (ranges of generated vibrations: force 400 N—in the case of sinusoidal vibrations and 311 N—in the case of random vibrations, max force without a fan 100 N; oscillation frequency range 2–2000 Hz; max acceleration 2.8 g in the case of sinusoidal vibrations and 2.0 g in the case of random vibrations; exciter mass 16 kg);
- TIRA signal amplifier P-Amp BAA 1000-2 TIRA V2 (maximum output 1250VA@4.0R);
- PCB impedance head series 2888D01 with a sensitivity of 102.1 mV/g (accelerometer) and 22.42 mV/N (force sensor);
- miniature, uniaxial, piezoelectric high sensitivity accelerometers PCB series 333B50 (measuring ranges: ±5 g, 0.5–3000 Hz ±5%)—applied for control of exciter’s vibration level;
- MODAL SHOP stinger, series 2110.

After the preliminary tests (regarding different ways of mounting the exciter), it was decided that the test stand would be based on a tripod with a gear column, two-section steel legs ended with rubber feet with spikes and a built-in spirit level. An aluminium guideway in the form of an elongated cylinder was screwed to the gear column. A steel plate with pre-drilled holes for the purpose of fixing the springs was screwed to the guideway from below. A steel ring was hung to the plate with the use of four springs. The exciter was screwed to the ring, and a steel connector was attached to the threaded mandrel of the exciter enabling mounting the stinger. The role of stinger is accurate axial transfer of loads from the exciter to the tested structure and limiting lateral constraint forces and moments. The impedance head was screwed to the other end of the stinger. In order to transfer dynamic load to the tested substrate, the impedance head was screwed to load plate.

**Figure 1:**
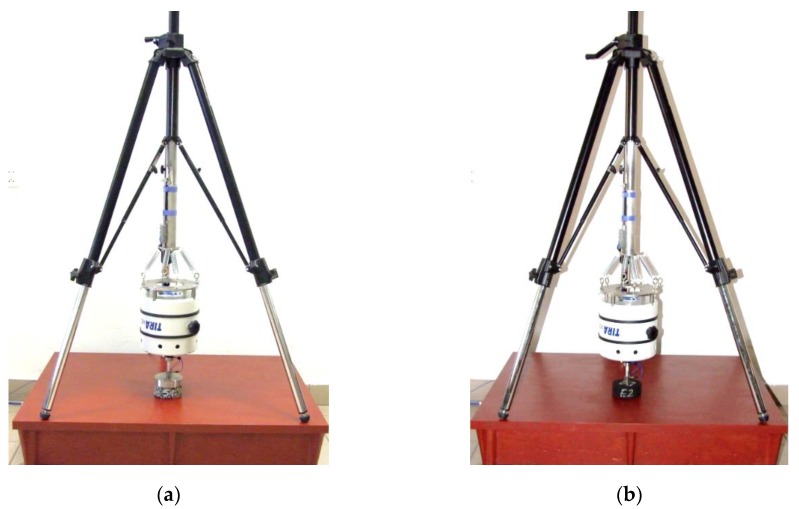
Test stand for measuring dynamic stiffness in laboratory and field conditions: (**a**) with massive load plate on the entire upper surface of the specimen; (**b**) with 30 mm diameter load plate.

**Figure 2:**
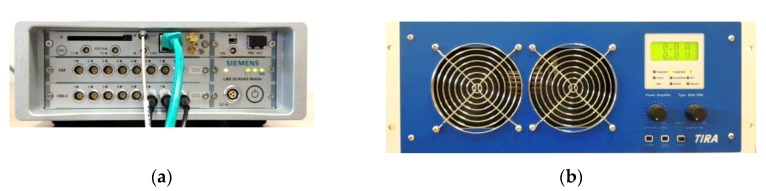
Multichannel 24-bit data acquisition hardware type SCADAS Recorder from SIEMENS (Plano, TX, USA) (**a**) and TIRA signal amplifier (**b**) (TIRA GmbH, Schalkau, Germany).

The constructed test stand and devices used to amplify and recording signals of force and acceleration are shown in Figure 1 and Figure 2.

Considering the impact of wearing course on the tyre/road noise [26] and the frequency range of sounds audible to humans (from about 16 Hz up to about 20 kHz [16]), it was decided that a much broader frequency range would be considered in this paper than in the case of typical dynamic diagnostics of building structures. Therefore, in the planned tests of dynamic stiffness of materials used for the wearing course, the frequency bandwidth up to 1024 Hz was adopted as significant. Assuming the same number of spectral lines, we obtain the Fast Fourier Transform (FFT) resolution of the recorded signal on the level of 1 Hz, which is perfectly sufficient considering the bandwidth, acquisition time of a single spectrum and the total measurement time (including up to a few hundred necessary spectra for later averaging).

According to ISO 7626-2 [23], with coherence of recorded signals at the level of 0.8 and a random error of 5%, a minimum of 178 averages are required to obtain 90% confidence in measurements. In the tests, 250 averages were adopted and time-domain weighting of the signals using the Hanning time–domain function recommended by ISO 7626-2 [23].

The recording and analysis of the data recorded for the purposes of this work were carried out using the dedicated LMS Test.Lab Spectral Testing software (version 16A, Siemens PLM Software, Plano, TX, USA).

In order to determine the impact of the load surface and the type and the amplitude of excitation on the level of dynamic stiffness of bituminous mixtures, control measurements were carried out on two samples with different characteristics: a rubberised asphalt mixture with a crumb rubber granulate (content 10.42% by weight; designated E1) and a mixture of stone mastic asphalt SMA8 (designated as S1). The samples had an identical diameter of 100 mm and a height of about 40 mm. Their characteristics are given in w Table 1.

Taking into account the size of aggregate grains used in bituminous mixtures, the load surface adopted by Vázquez and Paje [24] in measurements of the dynamic stiffness of samples by non-resonant method (circular plate with a diameter of 14 mm and surface area of approx. 1.54 cm^2^) was considered insufficient. Given the size of aggregate grains, and taking into account the mobility of the test stand and the maximum measuring ranges of the TIRA TV 51144IN exciter and the PCB 2888D01 impedance head, the load surface provided by a 30 mm diameter steel plate was considered a reasonable compromise.

The load transfer from the exciter to the sample was tested via both a circular plate with a 30 mm of diameter and a circular load plate that transfers the load to the entire surface of the sample (steel sheet 100 mm in diameter). Tests were carried out on a much stiffer bituminous mixture designated as S1 at the total static load of the sample of 5 kg and 10 kg.

The EN 29052-1 [22] and ISO 7626-2 standards [23] allow for both sinusoidal excitation (with constant amplitude and swept sinusoidal excitation frequency), and random (white noise) or impact excitations. In the initial measurements carried out on a sample of stone mastic asphalt (S1), random excitation in the form of white noise was tested, as well as another type in which the frequency of generated vibrations was steadily swept while maintaining a constant amplitude (so-called periodic chirp). The dynamic load amplitude was tested by changing the excitation level from 0.05 V to 0.20 V in increments of 0.05 V.

**Figure 3:**
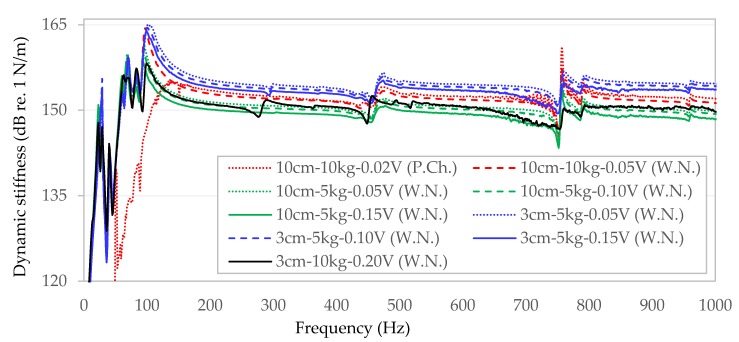
Dynamic stiffness over frequency for samples S1 considering varying load surface, static load level and vibration amplitudes (the legend used in brackets indicates the applied vibration excitation method: W.N.—white noise, P.Ch.—periodic chirp).

The results of measurements are shown in Figure 3. The values of dynamic stiffness on the vertical axis are expressed in N/m on the dB/Level scale (reference numerals used in the legend: 3 cm/10 cm—the diameter of the load plates; 5 kg/10 kg—the total static load of the specimens; 0.02−0.20 V—the signal amplification).

After comparing the results obtained from a test specimen from stone mastic asphalt S1 with random white noise excitation and a periodic chirp excitation, it was found that in the range of up to about 120 Hz, the dynamic stiffness values obtained from measurements vary significantly. With a periodic chirp excitation up to 120 Hz, the dynamic stiffness (red dotted line in Figure 3) after the initial increase decreases almost twice as much, compared to the dominant trend line. In excitations above 120 Hz, both types show similar trend lines.

In the analysis of the impact of the surface through which the dynamic load is transferred to the tested specimen, it can be seen that in the case of load on the entire surface (100 mm circular plate diameter) slightly lower values of dynamic stiffness of the tested sample are present (Figure 3—green lines), than in the case of load transmitted through a circular plate with a much smaller diameter of 30 mm (Figure 3—lines in blue).

Analysis of the results of the dynamic stiffness of the S1 sample obtained for different levels of excitation (Figure 3) indicates that the stiffness decreases with the increase of the dynamic amplitude generated by the exciter (Figure 3—dotted lines 0.05 V, dashed lines 0.10 V, solid lines 0.15 V and 0.20 V).

**Figure 4:**
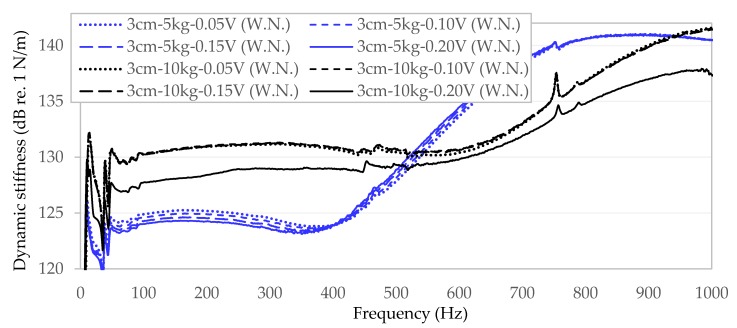
Dynamic stiffness over frequency for samples E1 considering varying static load level and vibration amplitudes.

A comparable effect of dynamic amplitude on dynamic stiffness was observed in the case of a much less stiff sample from a rubberised asphalt mixture marked as E1 (Figure 4). Unfortunately, in the case of this sample, a very significant impact of the static load (5 kg—blue lines, 10 kg—black lines) on the level of dynamic stiffness obtained by the specimen and the changeability of the trend line along with the increase in vibration frequency were found, which was not observed for a bituminous mix with significantly higher stiffness (S1).

The preliminary measurements clearly indicate that the level of static load has a much greater impact on the level and nature of the trend line of the tested dynamic stiffness of bituminous mixtures (especially in the case of less stiff materials) than the surface through which the dynamic load or the level of dynamic amplitude of generated vibration is transferred.

Following the results obtained as part of the initial measurements, in the following part of the study it was decided that we load the samples by using a 30-mm diameter load plate. This solution will allow for the application of dynamic loads with amplitudes close to the maximum force generated by the exciter without a fan connected (±100 N). After initial tests with various excitations (sinusoidal, periodic chirp, random, white noise, etc.), random excitation in the form of white noise was ultimately adopted. The parameters of the generated random signal were set in a slightly narrower band than recorded vibrations—i.e., from 10.2 Hz to 1000 Hz. The signal intensity after initial tests was set at 0.2 V, at which the permissible values of the exciter vibration accelerations and the amplitude of the force limited to ±100 N are not exceeded. With the applied static load from the exciter (at the level of about 10 kg) and a load/contact plate diameter of 30 mm, a test specimen static load at the level of 138.8 kPa was obtained. The tests were conducted in the temperature of 20 ± 1 °C.

## 3. Materials

The stiffness measurements in laboratory conditions were carried out using Marshall samples compacted in accordance with the standard BS EN 12697-30:2012 [27]. The bulk density of the tested bituminous mixture was determined on the basis of 3 specimens, and the coefficient of variation was less than 5%. The characteristics of the samples tested are compiled in Table 1.

The dynamic stiffness of bituminous mixtures was tested in two stages:

- Stage 1: on four individual samples with a diameter of about 100 mm and a height of about 40 mm, designated as: E1 (rubberised asphalt mixture, content of crumb rubber granulate 10.42% by weight, dry process), E2 (rubberised asphalt mixture, content of crumb rubber granulate 20.80% by weight, dry process), PA8 (porous asphalt) and S1 (SMA8);
- Stage 2: on twelve (three samples were made for each bituminous mixture) samples with a diameter of 100 mm and a height of about 60 mm, designated as: AC11 (asphalt concrete), S1 (SMA8), E1 (rubberised asphalt mixture, content of crumb rubber granulate 10.42% by weight, dry process), E2 (rubberised asphalt mixture, content of crumb rubber granulate 20.80% by weight, dry process).

The characteristics of the rubberised asphalt mixture (E1 and E2) and poroelastic layers of the PERS were similar.

In the case of E1 and E2 mixtures, the granulate was soaked with a vegetable liquefier (oil) before being added to the mixture.

The upper and lower layers of the samples were trimmed before testing in such a way as to guarantee the parallelism of these surfaces. No compensatory courses in the form of a thin layer of plaster of Paris and water were used, as was the case in other similar studies. In addition to the beneficial effect of a more even distribution of stresses in the test sample, this can cause additional damping that is difficult to estimate, and can even underestimate the dynamic stiffness of samples of very stiff composites. The tests in Stage 1 were conducted on single samples, and in Stage 2 on a larger number of samples in order to check the repeatability of results.

## 4. Result and Discussion

Dynamic stiffness measurements for each of the test specimens were made at three points (along the vertical axis and at two points shifted eccentrically with respect to axis of the sample). A total of 48 diagrams of dynamic stiffness of the specimens in a frequency–domain function were determined in Stages 1 and 2. At a constant excitation level of 0.2 V and with very different material compositions of the samples (from very flexible to very rigid), the force amplitudes obtained varied from 7 N (in low-stiffness samples) to 76 N (in very-stiff samples). Small force amplitudes were accompanied by much higher acceleration values, and vice versa—large force amplitudes were matched by significantly smaller values of vibration accelerations recorded on the exciter housing and in the impedance head. With the load/contact surface used, the amplitude of dynamic loads, depending on the type of specimen, oscillated from 9.9 kPa to 107.5 kPa.

**Figure 5:**
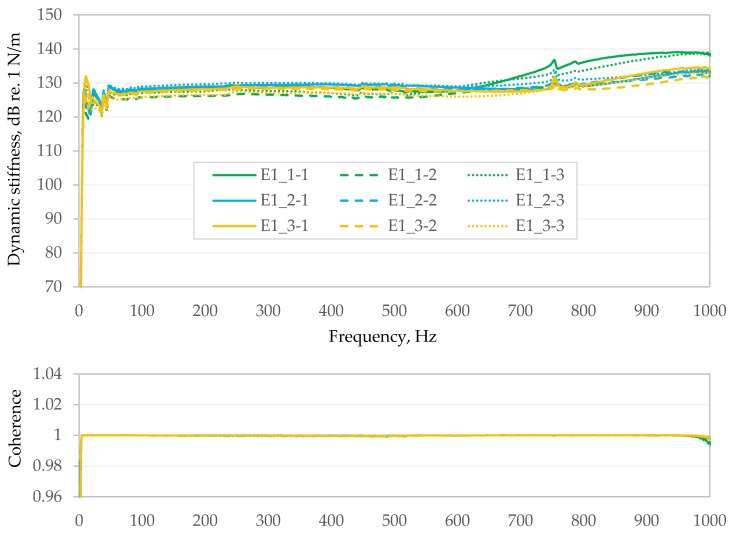
Dynamic stiffness (**top**) and coherence (**bottom**) of the E1 mixture.

**Figure 6:**
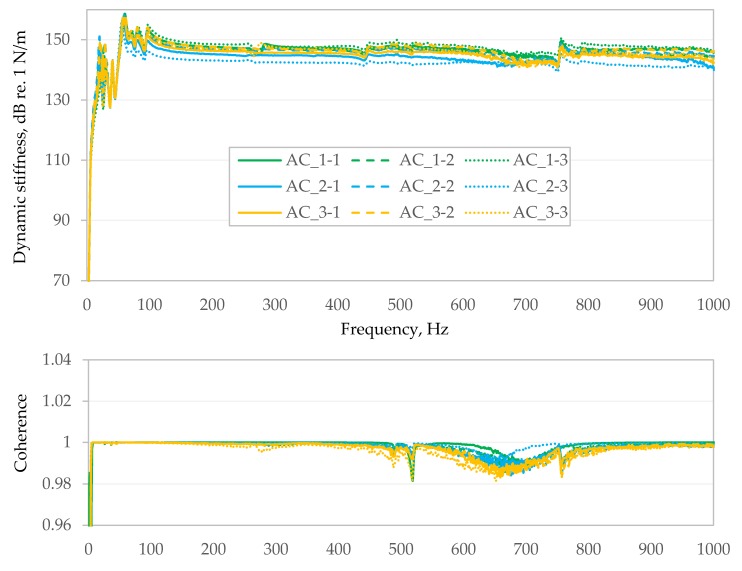
Dynamic stiffness (**top**) and coherence (**bottom**) of the AC mixture.

Figure 5 and Figure 6 summarizes results from the second stage of testing for samples made from E1 and AC bituminous mixtures. Each of the Figures presents the results of nine measurements. The upper graph shows averaged dynamic stiffness from over 250 spectra and is expressed in N/m on the dB/Level scale. The bottom graph shows the coherence between the excitation force and the recorded response (the ratio of the square of the magnitude of the cross-power spectrum between the excitation force (input) and the motion response of the structure (output), divided by the product of the input power spectrum times the output power spectrum [23]). In Figure 5 and Figure 6, it is presented linearly in the range from 0.96 to 1.04.

Coherence at a level close to 1.0 denotes a very good validation of test results. Local losses in coherence are typical for resonance or anti-resonance frequencies and can be caused by various types of factors [23]—among others, inadequate frequency resolution, inadequate time-domain weighting, and nonlinearities of the structure, inadequate force input or multiple input force. Momentary decreases in the coherence function may also occur in the case of resonance frequencies due to limitations related to inherent limitation of the exciter or at any other frequency due to nonlinearity within the structure. Low level of coherence in a wider band, in turn, may indicate a poor signal to noise ratio or an inadequate dynamic range.

After analysing the obtained graphs of coherence function (e.g., Figure 5), it should be stated that, in the case of less stiff bituminous composites such as E2 and E1, we experience a very good coherence (practically in the entire analysed frequency band at level 1.0—except for very low frequencies and frequencies close to 1 kHz).

In the case of much stiffer samples PA8, S1 and AC, coherence is not so perfect, but it is still well above the level of 0.95 (e.g., Figure 6).

**Figure 7:**
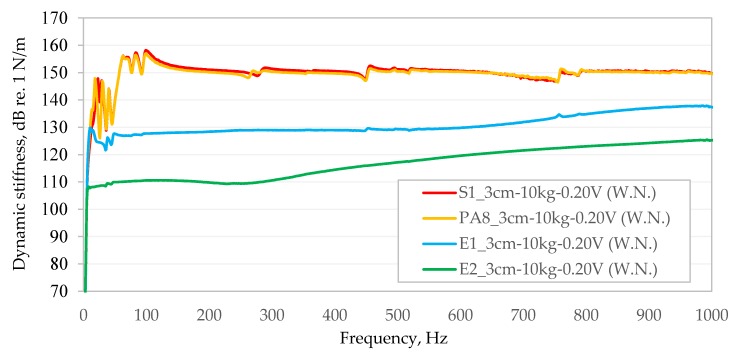
Comparison of dynamic stiffness of samples tested in the first stage.

**Figure 8:**
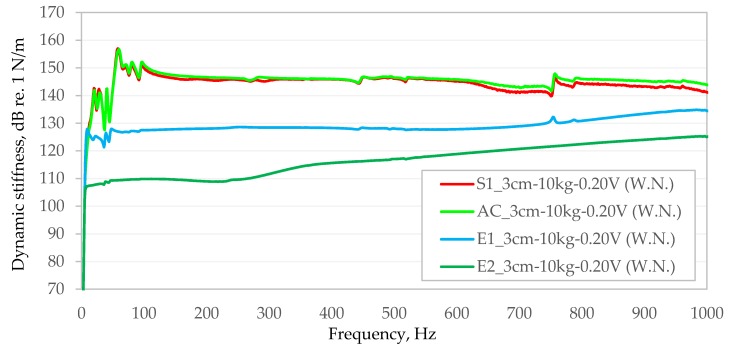
Comparison of dynamic stiffness of samples tested in the second stage.

In order to facilitate comparison of the dynamic stiffness obtained in the frequency–domain function with each other, the obtained results for individual bituminous mixtures were compiled in two graphs independently for each of the stages of the study in which the bituminous samples significantly differed in height (Figure 7 and Figure 8).

As can be seen in Figure 7 and Figure 8, the dynamic stiffness values obtained are not constant in the frequency band analysed in this article. For samples with lower stiffness, an upward trend in dynamic stiffness is visible with increasing frequency (E2 and E1). For example, in the case of the E2 bituminous mixture, dynamic stiffness in the range from 10 Hz to 1000 Hz increases non-linearly from 107.2 dB re. 1 N/m up to 125.3 dB re. 1 N/m. In the case of much more rigid bituminous samples (S1, PA8 and AC), except for the initial frequency band (from about 10 Hz to about 100 Hz), dynamic stiffness does not show a significant dependence on the frequency of generated vibrations.

The extreme values obtained from the tests are summarised in Table 2 and Table 3.

Figure 7 and Figure 8 show significant fluctuations in the dynamic stiffness of some bituminous mixture (S1, PA8 and AC) in the lower frequency range (up to about 100 Hz) and local trend line disturbances at higher frequencies. Disturbances in the trend line of dynamic stiffness of samples at higher frequencies at the level of a few hundred Hz are usually accompanied by local decreases in the value of the coherence function, indicating the occurrence of resonance frequencies of the tested system. The improvement of the process or smoothing of the dynamic stiffness function of test specimens in the frequency domain may be affected, to some extent, by covering any unevenness of the tested material before testing, for example, using a few millimetres-thin paste of plaster of Paris and water (as recommended by the standard [22]). However, this is rather an undesirable intervention of the tested material, which the authors of the paper would like to avoid in the consideration of the possibility of applying the proposed test methodology in real conditions directly on the upper surface of the wearing course of bituminous pavements. Further improvement of dynamic stiffness distributions of the tested samples can likely be obtained by using an unusual stinger made of a material much stiffer than that used in this study, for example, hard polyamide or steel. This will be the subject of further research by the authors.

## 5. Conclusions

The wearing course of the road pavement can be performed in various technologies. Porous asphalt, thin asphalt layers and poroelastic pavements are the preferred solution for reduction of tyre/road noise. In the case of poroelastic mixtures, their stiffness has a significant impact on the noise level of moving vehicles.

Dynamic stiffness tests are usually conducted in laboratory conditions, using dedicated devices like rheometers or servo-hydraulic fatigue machines. Tests of dynamic properties of road surfaces in field conditions are most often conducted with the help of modal hammers.

The results of carried out research in laboratory conditions showed that:

- The test stand built with a tripod, multichannel 24-bit data acquisition system, signal amplifier, vibration exciter, uniaxial piezoelectric accelerometers and a stinger attached to impedance head allows the measurement of dynamic stiffness of bituminous mixtures.
- The proposed method of measuring dynamic stiffness does not require the determination of the resonance frequency of the tested specimen and can be used both on various types of bituminous mixtures of varying shape and dimensions in laboratory, as well as directly on the wearing course of bituminous pavements.
- The dynamic stiffness of bituminous mixture is significantly affected by the load level. The constructed test stand allows us to conduct tests on the dynamic stiffness, taking into consideration the different levels of static and dynamic loads of the test specimens or the wearing course of the road pavements.
- The dynamic stiffness of the asphalt concrete, stone mastic asphalt layers and porous asphalt is significantly higher than the dynamic stiffness of poroelastic mixtures.
- Increasing the content of crumb rubber in the poroelastic layers reduces its stiffness which affects the reduction of tyre/road noise.

The presented test results should be considered as preliminary. It is necessary to conduct further tests using a stiffer stinger and different loading conditions (both static and dynamic) on samples under laboratory conditions and in field conditions on road surfaces.

## Figures and Tables

**Table 1:** Characteristics of the bituminous mixtures.

Bituminous Mixtures:		E1	E2	PA8	SMA8/S1	AC11/AC
Content, % by mass	Coarse aggregate	68.36	55.00	88.08	71.20	51.80
	Fine aggregate 0/2	—	—	1.87	11.55	30.48
	Limestone filler	11.59	13.95	3.75	10.15	12.12
	Cellulose fiber	0.46	0.40	0.30	0.30	—
	Binder	9.17	9.90	6.30	6.80	5.60
	Crumb rubber 1/4	10.42	20.80	—	—	—
Air void content, %		20.5	15.7	24.8	2.8	2.3
Bulk density, Mg/m^3^		1.614	1.513	1.856	2.386	2.444

**Table 2:** The extreme dynamic stiffness values in the 10–1000 Hz band—the first stage.

Bituminous Mixture	S1	PA8	E1	E2
MIN dynamic stiffness, dB re. 1 N/m	124.0	126.0	121.6	107.8
MAX dynamic stiffness, dB re. 1 N/m	158.2	157.0	137.9	125.4

**Table 3:** The extreme dynamic stiffness values in the 10–1000 Hz band—the second stage.

Bituminous Mixture	S1	AC	E1	E2
MIN dynamic stiffness, dB re. 1 N/m	124.1	123.2	121.3	107.2
MAX dynamic stiffness, dB re. 1 N/m	157.0	156.7	134.9	125.3
